# Impact of Pharmaceutical Compounds in the Bioremediation of Municipal Biosolids by the White-Rot-Fungi *Trametes hirsuta*

**DOI:** 10.3389/ffunb.2022.896043

**Published:** 2022-05-04

**Authors:** Sabrina Saibi, Lounès Haroune, Olivier Savary, Jean-Philippe Bellenger, Hubert Cabana

**Affiliations:** ^1^Universitéde Sherbrooke Water Research Group, Department of Civil and Building Engineering, Université de Sherbrooke, Sherbrooke, QC, Canada; ^2^Sherbrooke Pharmacology Institute, Université de Sherbrooke, Sherbrooke, QC, Canada; ^3^Department of Chemistry, Université de Sherbrooke, Sherbrooke, QC, Canada

**Keywords:** white-rot-fungi, municipal biosolids, laccase activity, pharmaceutical compounds removal, toxicity

## Abstract

The potential of microorganisms for the treatment of municipal biosolids is continuously growing. The present studies evaluated the potency of *Trametes hirsuta* for the reduction in biosolid mass, production of extracellular enzymes, and removal of pharmaceutical compounds (PhACs) in biosolid slurry in the presence and absence of spiked PhACs [5 non-steroidal anti-inflammatories (NSAIs) and 2 psychoactive compounds (PACs)]. Toxicity after 35 days of fungal treatment was also assessed. Results showed that the growth of *T. hirsuta* is limited above 25% and wholly inhibited above 50% of biosolids in the slurry. At 12% of biosolid concentration, biosolid mass was reduced by 90%, NSAIs were entirely removed, but PACs' removal was only ~20%. Increasing biosolid content to 25% did not markedly affect biosolid reduction but significantly enhanced the removal of PACs (>50%). Results also showed that both PhACs and biosolids induced the production of oxidative enzymes. In 12% biosolids in the slurry, the oxidative potential measured by the ABTS assay (O_ABTS_) reached 5,000 mM of O_ABTS_ in the presence of PhACs, and 2,500 mM of O_ABTS_ without PhACs, as compared to 1,200 mM of O_ABTS_ in control culture. Finally, we report that white rot fungi (WRF) treatment significantly decreased the toxicity of the biosolids.

## Graphical Abstract

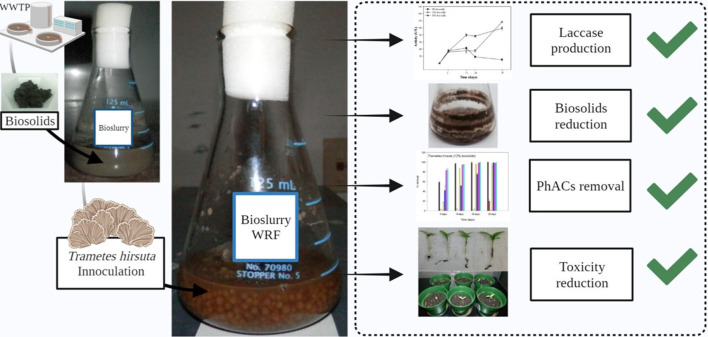

## Introduction

Biosolids production is increasing steadily worldwide due to stringent standards for waste water treatment and sludge disposal (Wang et al., 2008). Therefore, environmental friendly and economically sustainable management strategies of these large volumes of biosolids are needed for valorization (Hébert, 2015; Cano Londoño et al., 2017). Furthermore, these biosolids chiefly contain organic matter, nitrogen, phosphorous, and other nutrients, and their field valorization is economical and easy (Roy et al., 2011). Therefore, biosolids for soil amendments represent an opportunity to recover the essential nutrients for plant growth, restore soil, and reduce landfilling, and incineration of the resource (Torri and Lavado, 2009; Hébert, 2015). In addition, this approach could limit the use and production of chemical fertilizers (e.g., nitrates) and thus reduce their environmental impact.

A biosolid management strategy is to reduce biosolid volume and associated costs for disposal, storage, and transportation before valorization in agricultural fields (Shrestha et al., 2020). However, chemical methods traditionally used (Liu et al., 2017) suffer from the high cost of operation and the high-ecological risks during biosolid land spreading, limiting their application at large-scale. Biological treatments of biosolids are gaining interest as an alternative to overcome these limitations (More et al., 2010; Liu et al., 2017). Indeed, microorganisms such as filamentous fungi efficiently breakdown biosolid constituents and promote dewatering and settling (Fakhru'l-Razi et al., 2002). Another constraint in the use of municipal biosolids as fertilizer is that they contain potentially toxic chemicals (e.g., heavy metals), pathogens (Roy et al., 2011), and persistent organic pollutants such as pharmaceutical compounds (PhACs) in concentrations up to 100 μg/kg (Barcelo et al., 2010). The presence of PhACs can be potentially toxic to plants (Wu et al., 2015; Bartrons and Peñuelas, 2017) and could lead to contamination of the food chain through their uptake by the plants. For instance, lettuce (*Lactuca sativa*) has been reported to uptake a large amount of diclofenac and ibuprofen, up to 19 and 30 μg/kg, respectively (Calderón-Preciado et al., 2013). Both physicochemical and biological methods have been widely investigated to remove PhACs from biosolids (Weeks et al., 2000; Rodriguez-Rodriguez et al., 2011; Taha et al., 2018). Physicochemical methods generally achieve high efficiency for PhACs removal but they are often cost inefficient due to their high energy requirement (Luo et al., 2014). Biological methods are recognized as eco-friendly, energy, and cost-efficient approaches with wide application for the bioremediation process (Harms et al., 2011; Taha et al., 2018). White rot fungi (WRF), which are *Basidiomycota members*, have been identified as suitable organisms for their applications in environmental biotechnology (Lee et al., 2014) due to the low specificity of their extracellular (e.g., laccase lignin peroxidase, manganese-dependent peroxidase) and intracellular enzymes (e.g., cytochrome p-450) (Durairaj et al., 2016). They can transform a wide range of phenolic and non-phenolic compounds, such as PhACs, into an aqueous solution (Wong, 2009). Previous studies have shown that pharmaceutical compounds have a significant impact on the metabolism of WRF metabolism. Haroune et al. (2014) observed that a low concentration of pharmaceutical compounds in liquid medium induced a significant increase in laccase production by *Trametes hirsuta* and high efficiency for bioremediation NSAIs. Many studies aimed to improve the biological process in different culture conditions (Rodriguez-Rodriguez et al., 2011; Vasiliadou et al., 2016; Taha et al., 2018) and highlight the efficacy of filamentous fungi to growth in contaminated media. However, to the best of our knowledge, the impact of PhACs in the bioremediation of biosolids by WRF has not been investigated thoroughly till now.

The objective of the present studies was to investigate the impact of selected PhACs on the ability of *Trametes hirsuta* to grow in PhACs contaminated biosolid slurry, production of laccase, reduction in the biosolid volume, and removal of selected PhACs. Additionally, the toxicity of the fungal treatment was also addressed based on a seed germination assay.

## Materials and Methods

### Chemical Reagents

All chemicals used were of analytical and optima grades. Formic acid, methanol, and acetonitrile (Optima® grade for LC/MS) were purchased from Fisher Scientific (Ottawa, ON, Canada). Malt extract, yeast extract, D-glucose, and active pharmaceutical chemicals (acetaminophen, naproxen, ketoprofen, mefenamic acid, indomethacin, carbamazepine, and caffeine) of purity >95% were purchased from Sigma Aldrich (Saint-Louis, MO, USA).

### Municipal Biosolids

Municipal biosolids used for all experiments were obtained from a local wastewater treatment plant located in the province of Quebec, Canada. The physicochemical characteristics of biosolids have been presented in Supplementary Table S1. The targeted PhACs were not detected in the selected municipal biosolids.

### Fungal Strain

*Trametes hirsuta (IBB 450)* was obtained from the culture collection of the Institute of Biochemistry and Biotechnology, Tbilisi, Georgia. The strain was formulated in simple pellet mycelium form. The pelletization process was performed according to the method published by Haroune et al. (2014). The prepared blended mycelium suspension was used to obtain a growth under pellet form for the inoculation. All the experiments were conducted under sterile conditions (autoclaved 45 min at 121°C and 19 psi).

### Culture Conditions

To evaluate the efficiency of the selected fungal strain to reduce biosolid volume and remove selected PhACs, all experiments were carried out under sterile conditions to prevent interactions between fungi and endogenous bacteria that can affect treatment outcomes (Avella et al., 2014).

The two culture conditions, i.e., with and without PhACs load, were evaluated. The fungi *T. hirsuta* was grown on a rotary shaker at 135 rpm and 25°C in 250 ml Erlenmeyer flasks. For this, four treatments of municipal biosolids, 12, 25, 50, and 100% (w/v) were used. The biosolids were diluted with a culture medium containing 0.4% w/v glucose, 0.4% w/v yeast, and 1% w/v malt. The biosolid-based media were autoclaved (45 min at 121°C and 19 psi) before adding PhACs. Each flask contained 50 ml of the defined medium, 1 ml (120 mg_WRF_/g_inoculum_ dry weight of WRF) of blended mycelium suspension, and a final concentration of 16 ng/ml for each PhACs. The PhACs were added to the sterile bioslurry 72 h before the start of the experiment to allow compounds to reach equilibrium (sorption) with the matrix (Supplementary Figure S1). Bioslurry without PhACs was used as control.

The experiments were conducted for 35 days at 25°C. At regular time intervals of 5, 15, 20, and 35 days one triplicate of each concentration of biosolids was sacrificed (whole flasks), and PhACs were extracted from the bioslurry before the analysis (see Supplementary Details S1).

### Enzymatic Activity

Laccase (LAC) activity was quantified according to Touahar et al. (2014) by following the conversion of 0.5 mM of 2,2′-azino-bis- (3-ethylbenzthiazoline-6-sulfonic acid) (ABTS) to its radical cation (ABTS•^+^) at 420 nm (ε_max_ = 36,000 M^−1^ cm^−1^) in 0.1 M citric acid/0.1 M disodium hydrogen phosphate buffer at pH 3. One enzymatic activity unit (U) was defined as the amount of enzyme that transforms 1 μmol of substrate per minute. Activity measurements were carried out on a 96-well plate using a double-beam UV–Vis spectrophotometer (SpectraMax Plus 3250, Molecular Devices Corp., and Sunnyvale, CA). Additionally, the LAC production was also represented by the integral of the curve of the measured LAC activity to describe the variation of LAC activity in the media as a function of time. Defined by ${\text{O}}_{\text{ABTS}}=t\int_{0}^{t}f\left(t\right)dt$ where O_ABTS_ represents the total amount of ABTS oxidized in mM during the experimental timeline and t the time. Results report the mean of triplicates ± standard deviation.

### Gravimetric Measurement

The chemical oxygen demand (COD) was measured with the LR HACH kit (0–1,500 mg/L) according to US EPA (Method 8000) (HACH, 2014). Two (2) ml of the mixture was incubated at 150°C for 120 min in a preheated reactor (DRB 200 reactor). The COD was measured spectro photometrically at 350 nm. Total solids (TS), total dissolved solids (TDS), and total suspended solids (TSS) were assayed using Standard Methods part 2540 (Rice et al., 2017). Samples were measured in triplicate after 35 days of treatment (Supplementary Equations 1–3).

### Toxicity Assay

To compare the toxicity of treated and untreated biosolids, a seed germination test was conducted as per OECD guideline 208 (OECD, 2003). The test was performed using 10 seeds of tomatoes (*Solanum lycopersicum*) seeds/pot containing 100 g of soil and 0.3 g of biosolids. After10 days of seed germination, the percentage of relative root elongation (RE), relative seed germination (SG), and germination index (GI) were determined (Supplementary Equations 4–6).

### Pharmaceutical Quantification

Pharmaceutical compounds were analyzed during positive electro spray ionization (ESI+) source in multi-reaction-monitoring (MRM) mode on an Acquity UPLC XEVO TQ mass spectrometer (Waters Corporation, Milford, MA) equipped with an Acquity UPLC HSS-T3 column (100 mm × 2.1 mm, 1.8 μm) (Haroune et al., 2014) (Supplementary Table S2). The analytical method's limit and performance are presented in the Supplementary Table S3.

## Results and Discussion

With more than 50% of biosolids content, showed weak or no fungal growth. Thus, only results collected at 12 and 25% of biosolids concentration are presented.

### Laccase Activity in the Biosolid-Based Medium

**Figure 1:**
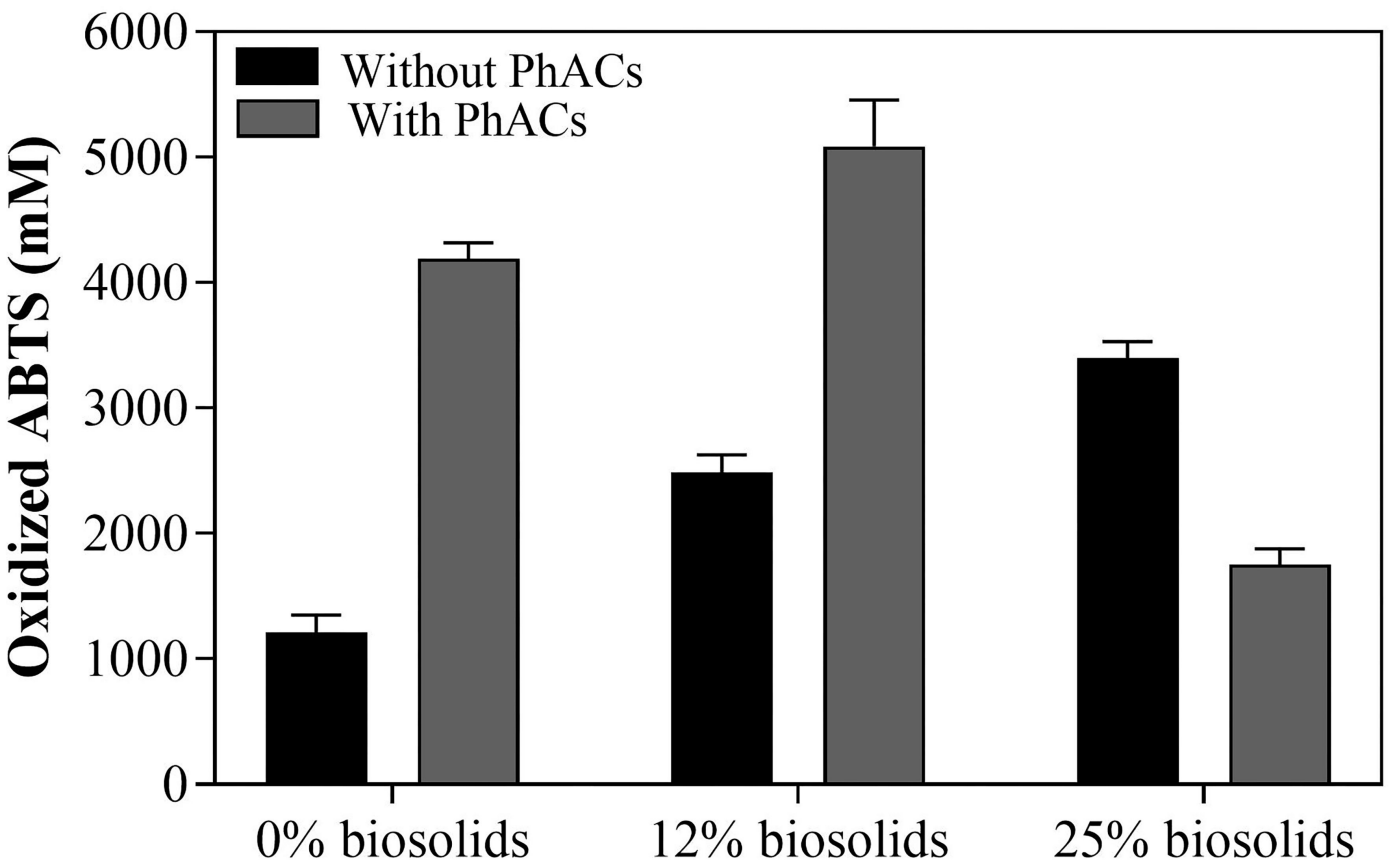
LAC production over the 35-day growth based on the daily enzymatic volumic activity with *T. hirsuta* in PhACs-free biosolid-based growth and in PhACs-contaminated biosolid-based growth.

An increase in biosolid concentration from 0 to 25%, in the absence of spiked PhACs, indicates that the amount of LAC produced range from 1,200 to 3,400 mM of O_ABTS_ (Figure 1); it suggests that that biosolid-based media is a suitable medium for enzyme production (Benítez et al., 2000). Furthermore, the presence of PhACs further stimulated LAC production in both 0 and 12% biosolid in the slurry (up to 5,000 mM of O_ABTS_). These results are consistent with the previous studies suggesting that PhACs could act as LAC inducers during PhACs treatment (Haroune et al., 2014) in liquid culture. On the contrary, at 25% biosolids in the slurry, LAC production was relatively lower with PhACs (1,750 mM of O_ABTS_) than without PhACs (3,400 mM of O_ABTS_) (Figure 1). LAC production was, however, still higher than in control (1,200 mM of O_ABTS_ at 0% biosolids and no PhACs). This illustrates that PhACs can promote LAC production but the response is influenced by the amount of biosolid in the slurry and the presence of PhACs. Nonetheless, these results show that LAC production can be enhanced by a wide variety of substrates (biosolids and PhACs) and that biosolids from municipal waste could be a suitable low-cost medium for affordable production of valuable oxidative enzymes. Consistent with its large spectrum of action (Russell and Yost, 2021; Unuofin et al., 2021), results show that *T. hirstuta* is suitable for LAC production in PhACs contaminated biosolid-based media.

### Biosolids Reduction

Reducing biosolid volume is essential in handling, storage, and overall resource management. Thus, the ability of the WRF strain to reduce biosolids volume was tested by monitoring the reduction of TS, TDS, TSS, and COD.

**Figure 2:**
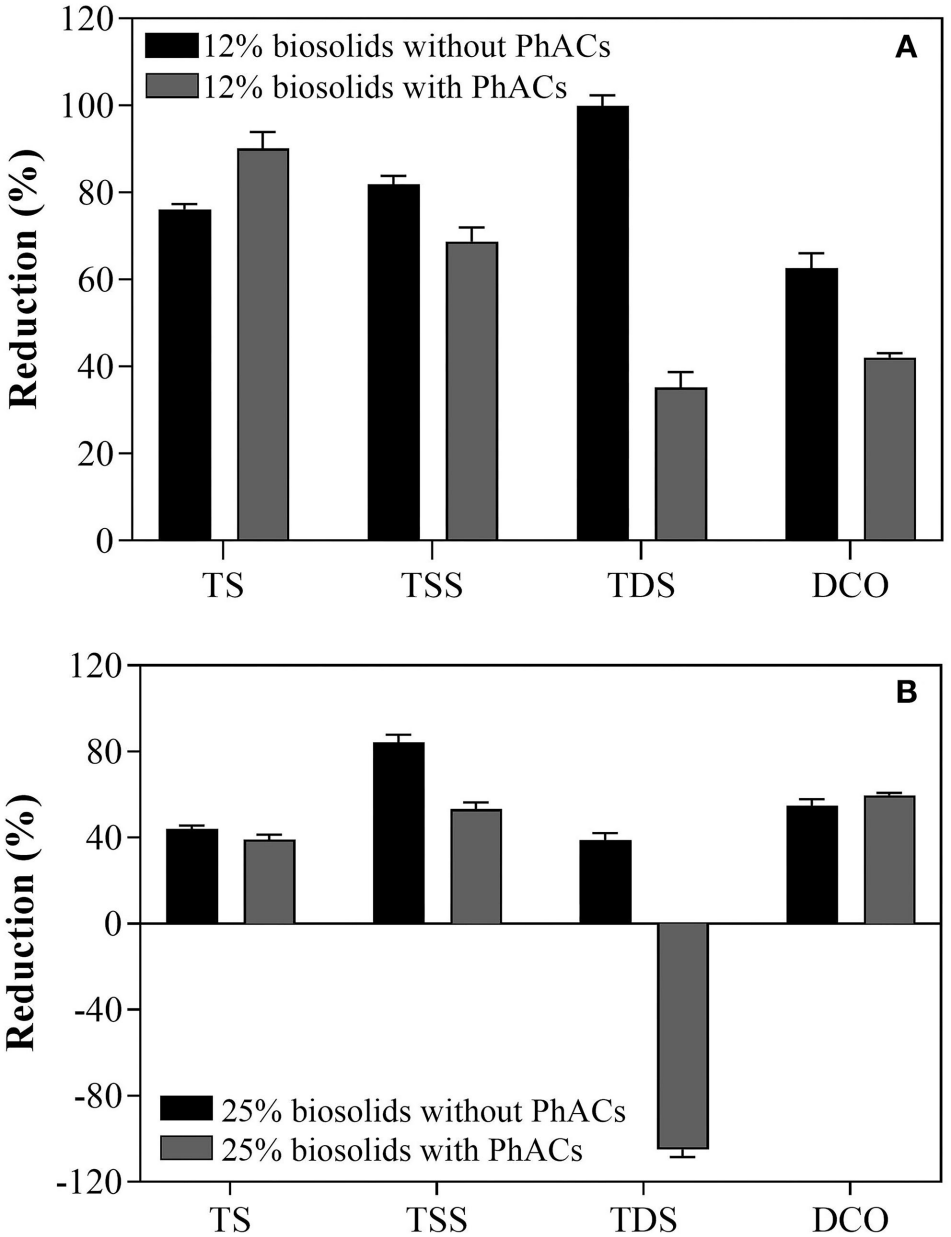
Biosolids volume reduction after 35 days of treatment with *T. hirsuta* in 12% biosolid-based growth **(A)** and in 25% biosolid-based growth **(B)**.

In the absence of PhACs in biosolids, reduction of up to 80% and close to ~50% in TS level was observed after 35 days interval in cultures containing 12 and 25% biosolids, respectively (Figures 2A,B). Further, the level of TSS was reduced by ~80% at both 12 and 25% biosolid concentrations. Furthermore, TDS was decreased by 90 and 40% in cultures containing 12 and 25% biosolids, respectively. Finally, the reduction of COD was observed to be ~60 and ~50% in 12 and 25% biosolid slurries, respectively. While results are likely to vary with the type of biosolid, and as reported here, efficacy is affected by slurry preparation (percentage of biosolids), these results show that WRF (*T. hirsuta*) treatment is a promising avenue for the ecofriendly reduction of biosolid volume. In the presence of PhACs, *T. hirsuta* maintained a robust efficacy to reduce biosolid volume, with efficient reduction of TS, TSS, and COD at 12 and 25% of biosolid in the slurry (Figures 2A,B). One notable effect of PhACs was observed on the efficacy to reduce TDS. Indeed, TDS significantly decreased at 12% of biosolid compared to the control (from ~100 to less than 40%). At 25% of biosolids, net production of TDS was observed (−100%).This latter result may be due to the solubilization of the volatile solids (VS) following the breakdown of the biosolids structure (Qiao et al., 2013). Alternatively, the high production of extracellular polymeric substances (EPS) by the WRF during treatment (Neumann et al., 2016) could also explain these observations. Overall, these results show the high efficacy of *T. hirsuta* strain to reduce biosolid volume potentially by directly using it as a carbon source (Harms et al., 2011).

White rot fungi can produce several extracellular enzymes (Harms et al., 2011), such as proteases, cellulase, lipase, and hydrolase (including laccase), that play a significant role in the reduction of biosolids (Roman et al., 2006; Shrestha et al., 2020). The treatment of biosolids with WRF culture offers several advantages over purified enzymes (Rouches et al., 2016). In addition, the use of several enzymes produced in the culture conditions improves the treatment efficacy (Roman et al., 2006); however, the purification of enzyme mixtures can be expensive and time-consuming. The direct use of WRF culture on biosolids allows for cost-efficient production of a wide array of enzymes (including intracellular enzymes), and their composition is tuned by the strain in response to the matrix characteristics and changes during the treatment.

### PhACs Removal

**Figure 3:**
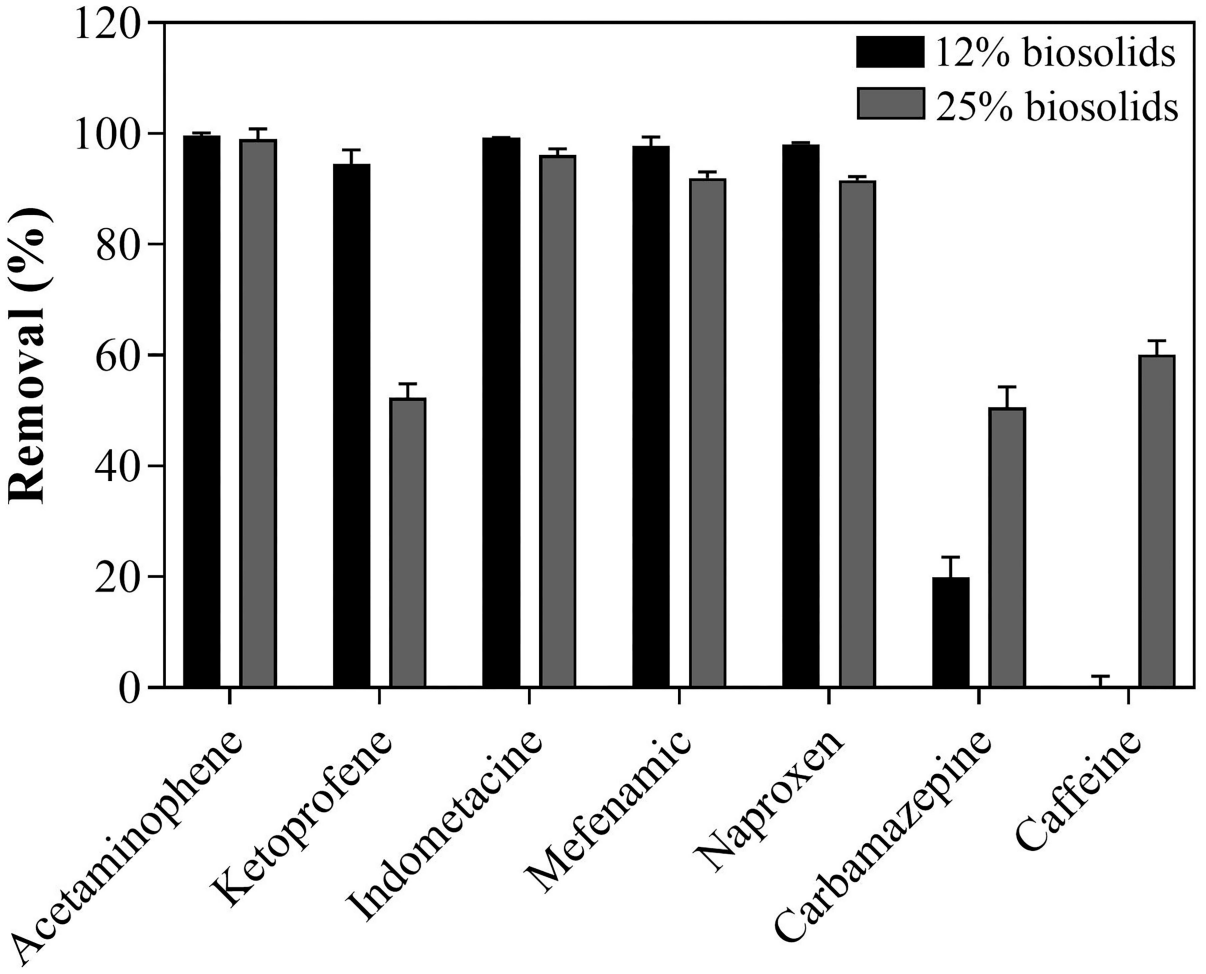
PhACs removal after 35 days of treatment with *T. hirsuta* in contaminated PhACs biosolids-based growth.

The removal efficiency of PhACs was molecule and medium dependent (% of biosolids). In cultures containing 12% biosolids, *T. hirsuta* achieved high removal efficiency (>80%) for all tested PhACs, except for PACs (carbamazepine and caffeine <20%) (Figure 3). The increase in biosolids content in the culture medium (from 12 to 25% biosolids) had contrasting effects, increasing the removal efficiency of some compounds (e.g., carbamazepine and caffeine), decreasing the removal of others (e.g., ketoprofen) (Figure 3), and having no effects on few (e.g., acetaminophen). These results are consistent with previous studies performed in defined liquid culture medium (Haroune et al., 2014, 2017), reporting high removal of NSAIs by *T. hirsuta*. There was no direct correlation between LAC activity (maximum at 12%, Supplementary Figure S2) and removal efficiency (maximum at 25%, Supplementary Figure S2). This result suggests that mechanisms not accounted for in the present studies could contribute to the removal of PhACs. Haroune et al. (2017) showed that the removal of ketoprofen was strongly correlated with the internalization of the compound before its transformation by intracellular enzymes. The decrease in ketoprofen removal with increasing biosolids concentration observed here could result from immobilization on biosolid interfering with internalization. Rodriguez-Rodriguez et al. (2011) reported a 50% elimination of carbamazepine by the WRF *T. Versicolor* with 25% of biosolid after 42 days of treatment, similar to results reported presently. Our data show that carbamazepine removal was biosolid concentration-dependent. The improved removal of carbamazepine (and caffeine) with higher biosolid concentration in the medium (Figure 3) could result from the immobilization of the compounds on biosolids (compounds—organic matter interactions) and cross-coupling reactions (intra- and inter-molecular interactions) (Avella et al., 2014). Although, more research is required to characterize the mechanism underlying PhACs removal in the biosolid-based growth medium. These results show that the selected WRF strain has great potential for reducing biosolid volume, but they can also efficiently reduce the PhACs load.

### Toxicity Assay

A germination test was performed with *T. hirsuta* treated slurry containing 12% biosolids which is the most efficient condition for PhACs removal, total solids reduction, and LAC production (Figures 1–3). The germination index (GI), relative root elongation (RE) and relative seed germination (SG) show a significant increase in the mixture containing biosolids treated with *T. hirsuta* strain as compared to untreated ones (Table 1). The root elongation and germination index increased more than 80% in treated biosolids compared to less than 24% in untreated biosolids. This reduced toxicity could result from fungal removal of toxic organic or inorganic compounds through active or passive uptake (Dhankhar and Hooda, 2011). The results are in agreement with Voběrková et al. (2017) who reported that the inoculation of the WRF *T. versicolor* in municipal solid waste led to an improvement in the compost quality and enhanced plant growth. Thus, plant growth promotion due to higher nutrient availability after fungal treatment cannot be ruled out. Nonetheless, these results illustrate that WRF treatment enhanced biosolid ability to support plant growth (thus toxicity reduction, improved nutrient availability, or both). While further investigation is needed, the present studies suggest that WRF fungi area suitable treatment of biosolid increasingly destined to be used as fertilizer in agricultural soils.

**Table 1:** Seed germination tests in biosolid amended soil.

**Sample**	**Relative seed germination (%)**	**Relative root elongation (%)**	**Germination index**
Treated biosolids	100 ± 2.62	82.6 ± 6.82	82.6 ± 6.82
Untreated biosolids	80.0 ± 1.86	23.1 ± 2.86	18.5 ± 2.29

*Biosolids were added without treatment (control) and after 35 days of treatment by T. hirsuta in a medium containing 12% biosolids.*

*Values reported are means of 5 replicates ± standard deviation*.

## Conclusion

For the first time, this study reports the presence of biosolid induced LAC activity during WRF bioremediation by *T. hirsuta*. LAC activity in biosolid slurries was modulated by the presence of PhACs, enhancing it at 12% biosolids but reducing it at 25% biosolids. However, LAC activity was higher than the control (0% biosolids, no PhACs), even in the latter condition. Moreover, the WRF treatment efficiently reduced biosolid volume, significantly reduced PhACs load, and markedly reduced biosolid toxicity to plants. Overall, these results show that biosolids could be a suitable low-cost medium for affordable LAC production and WRF treatment of biosolids is a promising avenue of research for reducing biosolid volume, organic contaminants removal, and toxicity reduction before application on agricultural soil.

## Data Availability Statement

The raw data supporting the conclusions of this article will be made available by the authors, without undue reservation.

## Author Contributions

SS: conceptualization, methodology, investigation, analysis, writing—original draft, and read and approved the final version of the article. LH: conceptualization, methodology, writing—review and editing, and read and approved the final version of the article. OS: laboratory and technical support. J-PB: writing—review and editing and read and approved the final version of the article. HC: supervision, project administration, writing—review and editing and read and approved the final version of the article. All authors contributed to the article and approved the submitted version.

## Funding

This work was supported by Discovery Grants from the Natural Sciences and Engineering Research Council of Canada (HC: 371681-2014 and J-PB: 386963-2011).

## Conflict of Interest

The authors declare that the research was conducted in the absence of any commercial or financial relationships that could be construed as a potential conflict of interest.

## Publisher's Note

All claims expressed in this article are solely those of the authors and do not necessarily represent those of their affiliated organizations, or those of the publisher, the editors and the reviewers. Any product that may be evaluated in this article, or claim that may be made by its manufacturer, is not guaranteed or endorsed by the publisher.
