# Supplementary material for: Impact of Pharmaceutical Compounds in the Bioremediation of Municipal Biosolids by the White-Rot-Fungi Trametes hirsuta
Source: Front Fungal Biol. 2022 May 4;3:896043. doi: 10.3389/ffunb.2022.896043 (PMC10512397; doi:10.3389/ffunb.2022.896043)
Supplement: Supplementary file 1 [file Data_Sheet_1.DOCX]

**Supplementary information**

**Impact of pharmaceutical compounds in bioremediation of municipal biosolids by the white rot fungi *Trametes hirsuta***

Sabrina Saibi*^1^,* Lounès Haroune*^3^**, Olivier Savary*^1^,* Jean-Philippe Bellenger*^2^,* Hubert Cabana*^1^**

## *^1^* *Université de Sherbrooke Water Research Group, Department of Civil and Building Engineering, Université de Sherbrooke, 2500 Boul. de l’Université, Sherbrooke, (Qc) J1K 2R1, Canada*

## *^2^Department of Chemistry, Université de Sherbrooke, 2500 Boul. de l’Université, Sherbrooke, (Qc) J1K 2R1, Canada*

## *^3^Sherbrooke Pharmacology Institute, Université de Sherbrooke, Campus de la santé, 3001 12 Ave N, Sherbrooke, (Qc) J1H 5N4, Québec, Canada*

* Address for correspondence:

Cabana Hubert, PhD

*Université de Sherbrooke Water Research Group,*

Department of Civil and Building Engineering, Université de Sherbrooke,

2500 boul. de l’Université, Sherbrooke, Québec, J1K 2R1, Canada

Tel: +1 (819) 821-8000, ext. 65457, Fax: +1 (819) 821-7974

Email: [Hubert.Cabana@USherbrooke.ca](mailto:Hubert.Cabana@USherbrooke.ca)

Lounès Haroune, PhD

*Sherbrooke Pharmacology Institute,*

Université de Sherbrooke, Campus de la santé,

3001 12 Ave N, Sherbrooke, (Qc) J1H 5N4, Québec, Canada

Tel: +1(819)821-8000, ext. 72491

Email: Lounes.Haroune@Usherbrooke.ca

**Table S1:** Physicochemical characteristics of the biosolid

| Parameters | Value | Unit |
| --- | --- | --- |
| pH | 7,37 | - |
| Total organic carbon | 30,5 | % |
| Total nitrogen | 5,20 | % |
| C/N | 5,86 | - |


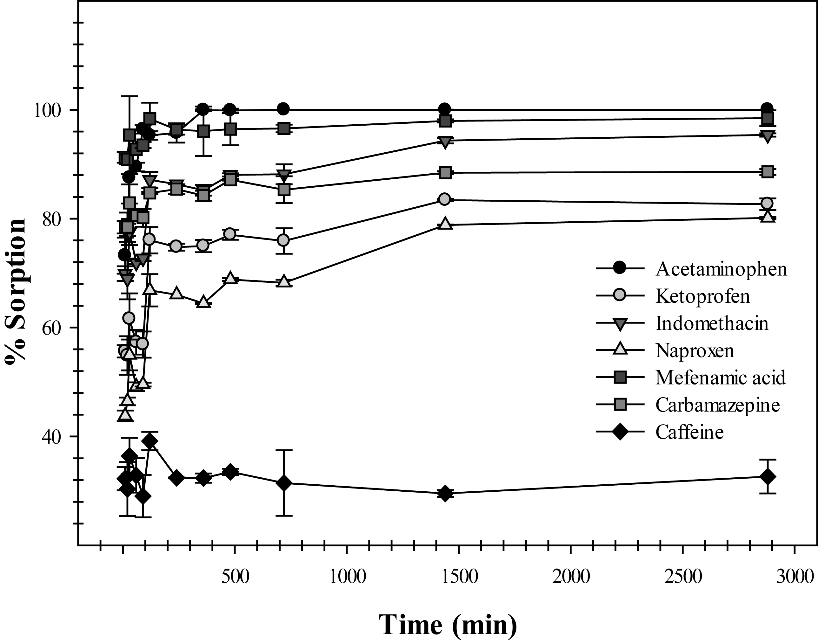


**Figure S1:** Percentage of sorption of the targeted PhACs


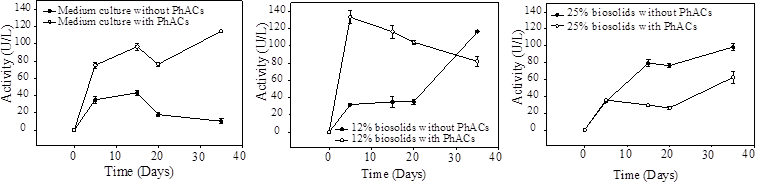


**Figure S2:** Time course of LAC activity in the different experiments

**Details S1: Extraction of PhACs from biosolids using a modified QuEChERS method (a manuscript related to the method validation is a part of separate article in preparation)**

Biosolid was transferred into a 50 mL falcon tube. 1 mL of acidic water (1% (v/v) formic acid) was added and samples were vortexed for 60 sec. 4 mL of ethyl acetate (1% formic acid) was added, and the solution was mixed until an emulsion formed (~ 60 sec). Then, 0.4 g of anhydride sodium sulfate, 0.1 g of sodium chloride and 0.4 g of ammonium acetate were added to the solution and immediately vortexed (60 sec) to avoid agglomeration of salt. The tubes were then centrifuged at 4300 xg for 20 min at 4°C. The organic layer (upper layer) was removed and evaporated to dryness under a gentle stream of N_2_ at 40°C (thermostated sand bath). The extracts were then dissolved in 1 mL of 100% acetonitrile (1% (v/v) formic acid), vortexed and sonicated for 30 sec. The mixture was transferred into a 1.5 mL Eppendorf tube containing the clean-up phase (50 mg C_18_/ 200 mg Na_2_SO_4_). The clean-up tube was swirled on a vortex mixer for 60 sec and centrifuged for 10 min at 20,050 xg at ambient temperature. Samples were evaporated to dryness and then reconstituted in 1 mL of a water/methanol mixture (1:1; v/v). Finally, solutions were filtered through a 0.2 µm PTFE filter syringe and analyzed by mass spectrometry (LC-MS/MS).

**Gravimetric measurement**

Total solids (TS) was determined following the Standard Method 2540 B (Rice et al., 2017) and equation (1) (1 mL of the mixture was dried to a constant weight in aluminum dishes at 105°C).

$TS (mg/L)=\frac{(A-B)\times1000}{sample volume ( mL)}$ eq.1

Where, A= weight of the dried residue + dish, in mg, and B= weight of the dish, in mg

Total dissolved solids (TDS) was determined following the Standard Method 2540 C (Rice et al., 2017) and equation (2). 1 mL of the mixture was filtered through a standard glass fiber filter and the filtrate was evaporated to dryness in a weighing dish and dried to a constant weight at 180°C.

$TDS (mg/L)=\frac{(C-D)\times1000}{sample volume (mL)}$ eq.2

Where, C= weight of the dried residue + dish, in mg, D= weight of the dish, in mg

Total suspended solids (TSS) were determined following the standard method 2540 D (Rice et al., 2017) and equation (3). 1 mL of the mixture was filtered through a standard weighing glass fiber and the residue retained on the filter was dried to a constant weight at 105°C.

$TSS (mg/L)=\frac{(E-F)\times1000}{sample volume (mL)}$ eq.3

Where, E= weight of the filter + dried residue, in mg, and F= weight of the filter, in mg

Chemical oxygen demand (COD) was measured with the LR HACH kit [0-1500 mg/L] according to US EPA (method 8000) (HACH, 2014). Two (2) mL of the mixture was incubated and heated at 150°C for 120 min in a preheated reactor (DRB 200 reactor). The COD was spectrophotometrically measured at 350 nm.

**Toxicity assay equation**

$RE=\frac{mean root length}{mean root length in control}\times100$ eq.4

$SG=\frac{seeds germinated}{seeds germinated in control}\times100$ eq.5

$GI=\frac{(SG)\times(RE)}{100}$ eq.6

**Table S2:** Mass spectrometry parameters used for the quantification of the selected PhACs. **(a manuscript related to the method validation is a part of separate article in preparation)**

| **Active Substance** | **Transition 1 : Quantification** | | | **Transition 2 : Qualification** | | |
| --- | --- | --- | --- | --- | --- | --- |
|  | Parent > T1  (m/z) | Cone  (V) | Coll  (V) | Parent > T2  (m/z) | Coll  (V) | Dwell time  (S) |
| Acetaminophen | 151.90 > 109.93 | 25 | 20 | 151.90 > 92.700 | 15 | 0.016 |
| Naproxen | 231.10 > 185.10 | 20 | 45 | 231.10 > 115.00 | 10 | 0.013 |
| Mefenamic Acid | 242.10 > 224.10 | 20 | 40 | 242.10 > 180.10 | 15 | 0.025 |
| Ketoprofen | 255.10 > 105.00 | 25 | 25 | 255.10 > 177.10 | 20 | 0.013 |
| Indomethacin | 358.20 > 139.00 | 25 | 45 | 358.20 > 174.20 | 20 | 0.013 |
| Carbamazepine | 237.13 > 194.10 | 25 | 35 | 237.13 > 179.05 | 30 | 0.025 |
| Caffeine | 194.98 > 137.98 | 35 | 25 | 194.98 > 109.95 | 20 | 0.016 |

**Table S3:** Limits and performances of the modified QuEChERS. **(a manuscript related to the method validation is a part of separate article in preparation).**

| Compounds | Recovery (%) | SD* (%) | LOQ (ng/g) |
| --- | --- | --- | --- |
| Acetaminophen | 96.3 | 4.66 | 1.21 |
| Caffeine | 80.4 | 1.13 | 0.82 |
| Carbamazepine | 82.8 | 3.57 | 2.07 |
| Ketoprofen | 96.4 | 1.74 | 0.88 |
| Mefenamic acid | 91.2 | 1.63 | 0.99 |
| Naproxen | 93.6 | 3.14 | 4.72 |
| Indomethacin | 94.8 | 2.40 | 0.84 |

*^*^Standard deviation*
